# Inhibition of the NLRP3 inflammasome by HSP90 inhibitors

**DOI:** 10.1111/imm.13267

**Published:** 2020-10-30

**Authors:** Sohaib Nizami, Kanisa Arunasalam, Jack Green, James Cook, Catherine B. Lawrence, Tryfon Zarganes‐Tzitzikas, John B. Davis, Elena Di Daniel, David Brough

**Affiliations:** ^1^ Alzheimer's Research UK Oxford Drug Discovery Institute University of Oxford Oxford UK; ^2^ Division of Neuroscience and Experimental Psychology School of Biological Sciences Faculty of Biology, Medicine and Health Manchester Academic Health Science Centre University of Manchester Manchester UK; ^3^ Lydia Becker Institute of Immunology and Inflammation University of Manchester Manchester UK

**Keywords:** caspase‐1, HSP90, inflammasome, inflammation, interleukin‐1, NLRP3

## Abstract

Excessive and dysregulated inflammation is known to contribute to disease progression. HSP90 is an intracellular chaperone known to regulate inflammatory processes including the NLRP3 inflammasome and secretion of the pro‐inflammatory cytokine interleukin(IL)‐1β. Here, primarily using an *in vitro* inflammasome ASC speck assay, and an *in vivo* model of murine peritonitis, we tested the utility of HSP90 inhibitors as anti‐inflammatory molecules. We report that the HSP90 inhibitor EC144 effectively inhibited inflammatory processes including priming and activation of NLRP3 *in vitro* and *in vivo*. A specific inhibitor of the β HSP90 isoform was ineffective suggesting the importance of the α isoform in inflammatory signalling. EC144 inhibited IL‐1β and IL‐6 *in vivo* when administered orally, and was brain‐penetrant. These data suggest that HSP90 inhibitors may be useful for targeting inflammation in diverse diseases that are worsened by the presence of inflammation.

## Graphical abstract

HSP90 inhibitors inhibit priming and activation of the NLRP3 inflammasome.

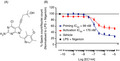

**Abbreviations**
ANOVA: analysis of variance
ASC: apoptosis‐associated speck‐like protein containing a caspase recruitment domain
ATP: adenosine triphosphate
BMDM: bone marrow‐derived macrophage
BSA: bovine serum albumin
DAMP: damage‐associated molecular pattern
DMEM: Dulbecco's modified Eagle's minimal essential medium
ELISA: enzyme‐linked immunosorbent assay
HSP: heat‐shock protein
IL: Interleukin
LDH: lactate dehydrogenase
LPS: lipopolysaccharide
NF: nuclear factor
NLRP3: NACHT, LRR and PYD domain‐containing protein 3
PAMP: pathogen‐associated molecular pattern
PBS: phosphate‐buffered saline
PBS‐T: phosphate‐buffered saline, 0·1% Tween‐20
PenStrep: penicillin and streptomycin
SEM: standard error of the mean
TLR: Toll‐like receptor

## INTRODUCTION

Inflammation is a protective host response to pathogens, yet is known to be destructive and damaging when excessive. In such contexts, inflammation is considered to be a disease‐modifying process and there are significant attempts underway to target damaging inflammatory responses occurring in diseases such as COVID‐19,^1^ and in other disease conditions.^2^, ^3^, ^4^ At the heart of damaging inflammatory responses in many diseases is a multimolecular complex called the NLRP3 inflammasome.^5^

NLRP3 (NACHT, LRR and PYD domain‐containing protein 3) is an important component of the innate immune system where it acts as a pattern recognition receptor (PRR) in cells such as macrophages. NLRP3 senses pathogen‐ or damage‐induced cellular stress and oligomerizes with an adaptor protein called ASC (apoptosis‐associated speck‐like protein containing a CARD) to form an inflammasome complex, which catalyses the activation of the protease caspase‐1, which then subsequently cleaves pro‐inflammatory cytokine precursors interleukin(IL)‐1β and IL‐18 to active forms that are then released.^6^ Caspase‐1 also cleaves the pore‐forming protein gasdermin D leading to pores in the cell membrane and pyroptotic cell death,^7^, ^8^ which allows the release of inflammasome complexes into the extracellular space where they further propagate inflammation,^9^, ^10^ and in models of Alzheimer's disease where extracellular ASC specks seed amyloid deposition and aggregation.^11^

There is thus great interest in developing inhibitors for the NLRP3 inflammasome.^5^, ^12^ Heat‐shock protein 90 (HSP90) is an abundant protein involved in a wide variety of cellular signalling processes, including inflammation, by virtue of it binding to and regulating diverse client proteins within cells.^13^ HSP90 is suggested to bind NLRP3, and regulate inflammasome activation^14^, ^15^ and IL‐1β secretion.^16^ Here, we report the use of HSP90 inhibitors to inhibit NLRP3‐dependent inflammation and propose this class of molecules as interesting candidates for studying inflammasome responses in disease, and highlight the potential for repurposing clinical HSP90 inhibitors to target inflammation.

## MATERIALS AND METHODS

### Compounds

BIIB021 (Cat No: 4608), EC144 (Cat No: 4701) and MCC950 (Cat No: 5479) were purchased from Tocris. TAS‐116 (Cat No: DC8142) was purchased from DC Chemicals. Onalespib (Cat No: HY‐14463) was purchased from MedChemExpress. KUNB31 (Cat No: SML2273) was purchased from Merck.

### BMDM cell culture

Immortalized bone marrow‐derived macrophages (iBMDMs) stably expressing ASC‐mCherry were previously described.^17^ ASC‐mCherry BMDMs were cultured in DMEM (high glucose, GlutaMAX, pyruvate, Thermo Fisher Scientific, Loughborough, UK), supplemented with 10% (v/v) heat‐inactivated (HI) FBS (Thermo Fisher Scientific) and 1% (v/v) penicillin–streptomycin (Thermo Fisher Scientific). Cells were seeded into 384‐well plates at 10 000 or 30 000 cells per well for the speck imaging or for the caspase‐1 experiments or seeded into 6‐well plates at 2 million cells per well for IL‐1β and LDH assays. Primary BMDMs were isolated and cultured as previously described.^17^ In brief, bone marrow cells were isolated from wild‐type C57BL6/J mice, and red blood cells were lysed and then cultured for 7 days in 70% DMEM (containing 10% v/v FBS and 1% v/v penicillin–streptomycin) supplemented with 30% L929 mouse fibroblast conditioned media. Before experiments, BMDMs were seeded overnight into 24‐well plates at 500 000 cells per well.

### ASC speck assay in iBMDM cells

iBMDMs were seeded into 384‐well plates using a FlexDrop bulk liquid handler and left to settle overnight. The compounds were dispensed into 384‐well plates using the Echo 550 acoustic liquid handler one day prior to experiment at various concentrations. The PerkinElmer Janus automated liquid handler was used to add treatment media (DMEM with no phenol red, Thermo Fisher Scientific, 21063029) containing lipopolysaccharide (LPS, from *Escherichia coli* O55:B5, Sigma‐Aldrich, L2637) (1 µg ml^−1^) and a nuclear stain, DRAQ5 (0·1 µM, Thermo Fisher Scientific), to the plates containing compounds. The LPS and compound mix were transferred to the cell plates and incubated for 2 h (priming). Nigericin (10 µM) was subsequently added for an additional 2 h to activate NLRP3. In the NLRP3 activation experiment, LPS (1 µg ml^−1^) and the nuclear stain, DRAQ5 (0·1 µM, Thermo Fisher Scientific), were added to the cells for 2 h, followed by media change with treatment media containing EC144 or MCC950. After 15‐min compound incubation, nigericin (10 µM) was added for an additional 2 h. The experiments were terminated by adding paraformaldehyde (final 2%) without any wash steps, and after 30 min, the plates were imaged using the GE IN Cell Analyzer 6000 using a 10×/0·45 Plan Apo, CFI/60 objective, 2 fields of view. Images were analysed using the IN Cell Developer Toolbox 1.9.2 software. The main steps of the analysis protocol were as follows: segmentation of the nuclei using the DRAQ5 channel, segmentation of the ASC specks using the mCherry channel, dilation of the nuclear mask to generate a pseudo‐cell region, and then target linking of ASC specks within this cell region. The total number of cells and the number of cells with ASC specks were calculated; from this, the percentage of cells with specks were calculated.

### NLRP3 inflammasome assays

#### In vitro

ASC‐mCherry iBMDMs were stimulated with LPS (1 µg ml^−1^) in treatment media (as described above) for 2 h. Compounds were also added at the same time as LPS addition (priming experiment) (final DMSO concentration 0·1%). After 2‐h incubation, nigericin (10 µM) was added and further incubated for 2 h. The supernatants were harvested for the quantification of IL‐1β by ELISA (R&D Systems, DY401, Abingdon, UK), cell death by LDH release (Thermo Fisher Scientific, 88953) or caspase‐1 activity by a caspase‐1 Glo assay (Promega, G9951, Southampton, UK). The assays were carried out following the manufacturer's instructions. PHERAstar FSX plate reader was used to measure absorbance/luminescence.

For the experiments with primary BMDMs, cells were incubated in serum‐free DMEM and treated with EC144 (1 µM) or DMSO (0·1% v/v) for 15 min prior to the addition of LPS (100 ng ml^−1^ or 1 µg ml^−1^) for 4 h. Supernatants were collected, and cells were lysed (150 mM NaCl, 50 mM Tris–HCl, 1% v/v Triton‐X‐100, pH 7·3, supplemented with a protease inhibitor cocktail). IL‐6 release was determined in the supernatant by ELISA (DY406, R&D Systems) according to the manufacturer's instructions. NLRP3, IL‐1β and HSP70 levels were assayed by Western blotting of the cell lysates. Proteins were separated by Tris–glycine SDS–PAGE and transferred onto nitrocellulose membranes. Membranes were blocked (1 h, RT) in 5% w/v milk in PBS with 0·1% v/v Tween‐20 (PBS‐T) before incubation (overnight, 4°) with antibodies targeting either NLRP3 (Cryo2, Adipogen, Liestal, Germany), IL‐1β (AF‐401, R&D) or HSP70 (4872, Cell Signaling Technology, Waltham, MA). Membranes were washed three times for 5 min in PBS‐T before incubation with appropriate HRP‐conjugated secondary antibodies. Membranes were washed a further three times before visualization using Amersham ECL Prime detection agent and captured using G:Box Chemi XX6 (Syngene, Cambridge, UK). Membranes were reprobed for β‐actin (AC‐15, HRP‐conjugated, Sigma, Poole, UK) to ensure equal protein loading.

#### In vivo

All procedures were performed with appropriate personal and project licences in place, in accordance with the Home Office (Animals) Scientific Procedures Act (1986), and approved by the Home Office and the local Animal Ethical Review Group, University of Manchester. Male C57BL6/J mice (Charles River), 8–10 weeks old, were administered either a vehicle control (0·1% Tween‐80 (v/v), 0·5% carboxymethylcellulose (w/v) in PBS), MCC950 (20 mg kg^−1^) or EC144 (0·1, 1, 10, 20 and 30 mg kg^−1^) by oral gavage before intraperitoneal (i.p.) injection of 1 µg LPS (*Escherichia coli* 0127:B8, L3880, 2 µg ml^−1^, 500 µl). After 2 h, mice were anaesthetized (induced at 3% in 33% O_2_, 67% NO_2_, maintained at 1–2%) before i.p. injection of ATP (100 mM, pH 7·4, 500 µl) or phosphate‐buffered saline (PBS) control for 15 min. The peritoneum was then lavaged and blood collected via cardiac puncture. Mice were then transcardially perfused with cold PBS, and the brain was collected and snap‐frozen on dry ice. The peritoneal lavage was used for cytokine analysis of IL‐1β and IL‐6 by ELISA (R&D Systems; IL‐1β DY401, IL‐6 DY406).

PK analysis was performed on brain samples and on plasma isolated from the blood. Briefly, control plasma was spiked to create calibration standards according to the previous experiments. To each standard, 200 µl IS (Internal Standard solution: 500 ng ml^−1^ tolbutamide (Sigma, T0891) in acetonitrile (Fisher, 10630131)) was added. To 50 µl aliquots of sample plasma, 5 µl of 1:1 acetonitrile:water and 200 µl IS were added. Blanks were prepared by adding 5 µl of 1:1 acetonitrile:water and 200 µl IS to 50 µl control plasma. These samples were mixed (150 rpm, 15 min) and centrifuged (1700 × *g*, 15 min). 50 µl of the resulting supernatant was added to 100 µl water, and samples were mixed (100 rpm, 15 min). Tissues were weighed, and 1 ml g^−1^ water was added. ZrO_2_ beads (ZrOB10, Web Scientific) were added, and the tissue was homogenized in a bullet blender (Next Advance BBX24B). Blank tissue homogenates were prepared in the same way as the samples. Blank homogenate was spiked to create calibration standards according to the previous experiments and then treated as for plasma. Samples were analysed by UHPLC‐ToF mass spectrometry using electrospray ionization utilizing an Agilent 1290 UHPLC/Agilent 6550 QToF equipped with an Acquity BEH Phenyl (50 × 2·1) mm, 1·7 µm column.

### Data presentation and analysis

**Figure 1:**
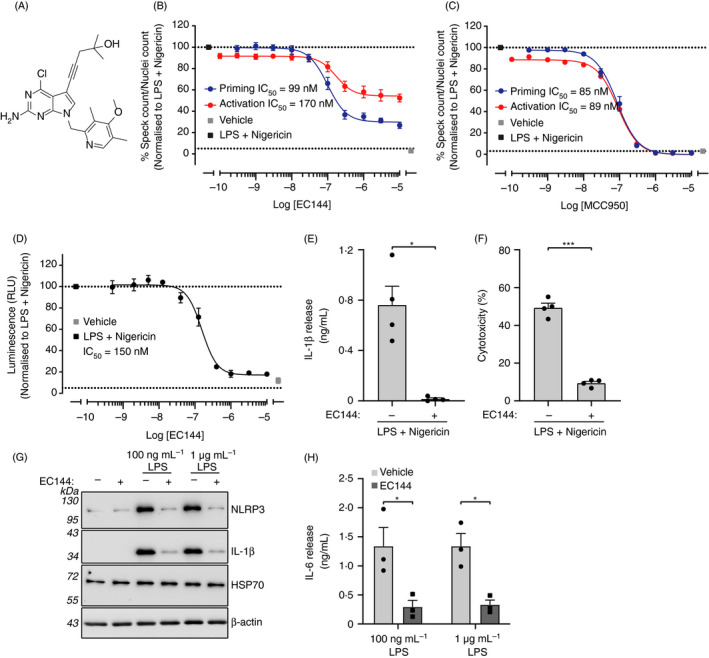
EC144 inhibits NLRP3 in iBMDMs. (A) Chemical structure of EC144. (B) ASC‐mCherry iBMDMs were treated with indicated concentrations of EC144 and LPS (1 µg ml^−1^) for 2 h followed by nigericin treatment (10 µM, 2 h) after which PFA was added (blue trace). In a parallel experiment, EC144 was added after LPS treatment just prior to nigericin (red trace). Percentage of ASC specks/total nuclei normalized to LPS and nigericin are presented. Data are the mean ±SEM of 3 independent experiments with each separate measurement made in duplicate. (C) The experiment described in B was repeated except using MCC950 instead of EC144. (D) ASC‐mCherry iBMDMs were treated with EC144 at the same time as LPS (1 µg ml^−1^, 2 h) and subsequently stimulated with nigericin (10 µM, 2 h), after which supernatants were analysed for caspase‐1 activity using caspase‐1 Glo. Data are presented as the mean ±SEM of 3 independent experiments with each separate measurement made in duplicate. (E) ASC‐mCherry iBMDMs were treated with EC144 (10 µM) and LPS (1 µg ml^−1^) together for 2 h before treatment with nigericin (10 µM, 2 h), after which supernatants were analysed for IL‐1β cytokine release using ELISA. Data are presented as the mean +SEM of four separate experiments with each separate measurement made in duplicate. Data were analysed using an unpaired *t*‐test with Welch's correction, *P < 0·05. (F) ASC‐mCherry iBMDMs were treated with EC144 (10 µM) and LPS (1 µg ml^−1^) together for 2 h before treatment with nigericin (10 µM, 2 h), after which supernatants were analysed for LDH release. Data are presented as the mean +SEM of four separate experiments. Data were analysed using an unpaired *t*‐test with Welch's correction, ***P < 0·001. (G) Primary BMDMs were treated with EC144 (1 µM) for 15 min before treatment with LPS (100 ng ml^−1^ or 1 µg ml^−1^, 4 h). Cell lysates were Western‐blotted for NLRP3, IL‐1β and HSP70. Representative blots from three experiments are shown. (H) IL‐6 release detected in the supernatants of cells treated in G, determined by ELISA. Data are presented as the mean +SEM of three experiments. Data were analysed using a two‐way ANOVA with post hoc Sidak's test. *P < 0·05.

**Figure 2:**
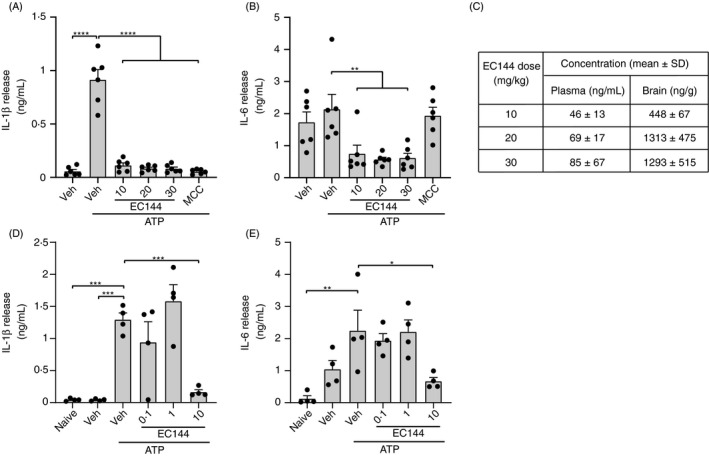
Oral administration of EC144 inhibits cytokine production in a murine model of peritonitis. (A–C) Mice were administered with either a vehicle control, EC144 (10, 20, or 30 mg kg^−1^) or MCC950 (MCC, 20 mg kg^−1^) by oral gavage immediately prior to i.p. injection of LPS (1 µg). 2 h after LPS injection, mice were anaesthetized before i.p. injection of ATP (100 mM, 500 µl, 15 min) or vehicle control. IL‐1β (A) and IL‐6 (B) in the peritoneal lavage were detected by ELISA (*n* = 6). (C) Pharmacokinetic analysis of EC144 concentration in the plasma and brain from animals treated in A and B (*n* = 3). (D,E) Mice were administered with either a vehicle control or EC144 (0·1, 1 or 10 mg kg^−1^) by oral gavage and treated the same as in A‐C. IL‐1β (D) and IL‐6 (E) in the peritoneal lavage were detected by ELISA (*n* = 4). Data are presented as the mean ±SEM. *P < 0·05, **P < 0·01, ***P < 0·001, ****P < 0·0001 as determined by a one‐way ANOVA with Dunnett's (vs vehicle +ATP control) post hoc analysis.

Data were analysed using the Prism software V7. Mean ±SEM were calculated, as indicated. Non‐linear regression fit was used to generate the IC_50_. Data were analysed using an unpaired *t*‐test with Welch's correction (Figure 1E,F), or a two‐way ANOVA with post hoc Dunnett's and Sidak's test (Figure 1H), or a one‐way ANOVA with Dunnett's post hoc analysis (Figure 2). A *P* value ≤0·05 was considered statistically significant in all experiments (*P ≤ 0·05, **P ≤ 0·01, ***P ≤ 0·001 and ****P ≤ 0·0001).

## RESULTS AND DISCUSSION

Previous studies using natural product‐derived HSP90 inhibitors, such as carnosol, geldanamycin or its analogue 17‐DMAG, have suggested that HSP90 modulates inflammation partly via NLRP3 inhibition.^14^, ^18^, ^19^ As well‐characterized synthetic HSP90 inhibitors, some of which are in clinical trials, are commercially available, we decided to test a number of structurally distinct HSP90 inhibitors in NLRP3 inflammasome assays. Initially, we used an NLRP3 inflammasome assay based on the formation of ASC specks in an ASC‐mCherry‐expressing mouse iBMDM cell line.^17^ Activation of the canonical NLRP3 pathway is typically achieved with two steps: a priming step to induce NF‐kB activation and transcription of NLRP3 and pro‐IL‐1β; and an activation step that results in inflammasome formation and caspase‐1 activation. As HSP90 is known to be involved in inflammatory signalling,^13^ to achieve the maximal effect against an NLRP3 response we tested HSP90 inhibitors against the overall canonical NLRP3 response by dosing them at the same time we initiated priming with LPS. BIIB021,^20^, ^21^ onalespib^22^, ^23^ and TAS116,^24^ which are reported to be potent HSP90 inhibitors, inhibited ASC speck formation induced by LPS (1 µg ml^−1^, 2 h) and nigericin (10 µM, 2 h) treatment with IC_50_ values of 80, 100 and 500 nM, respectively. Interestingly, KUNB31,^25^ which preferentially inhibits only one of the four HSP90 family members, the β isoform, which is constitutively expressed in the cytoplasm, was inactive up to 10 µM, suggesting that inhibition of the α isoform of HSP90 mediates the inhibitory effects on the NLRP3 response (Table 1).

**Table 1:** HSP90 inhibitors inhibit NLRP3‐dependent ASC speck formation in iBMDMs

Commercial name	Compound structure	CAS number	Speck IC_50_	Selectivity		Ref
				HSP90α	HSP90β	
BIIB021	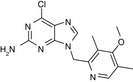	848695‐25‐0	80 nM	Non‐isoform selective		20
Onalespib	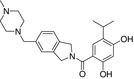	912999‐49‐6	100 nM	Non‐isoform selective		23
TAS‐116	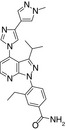	1260533‐36‐5	500 nM	Non‐isoform selective		24
KUNB31	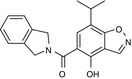	2220263‐80‐7	N.D.	β isoform selective		25

Provided are the chemical structures of HSP90 inhibitors, BIIB021, onalespib, TAS‐116 and KUNB31. ASC‐mCherry iBMDMs were treated with LPS (1 µg ml^−1^) and the inhibitor (2 h) followed by nigericin (10 µM, 2 h) as described in Methods, and the IC_50_ for ASC speck inhibition is provided. Data are the mean of 6 (BIIB021), 3 (onalespib) and 2 (TAS‐116, KUNB31) independent experiments.

Because of the strong inhibitory effect against inflammasome assembly observed with BIIB021, we decided to source a BIIB021 derivative, EC144, which is a second‐generation BIIB021 HSP90 inhibitor (Figure 1A).^26^ In the ASC speck assay, EC144 inhibited LPS and nigericin induced ASC speck formation with an IC_50_ of 99 nM (Figure 1B). In order to understand whether the compound worked in the NLRP3 priming or activation stage, we carried out a parallel experiment where EC144 was added after LPS before nigericin. In this case, EC144 was less potent and inhibited ASC speck formation with an IC_50_ of 170 nM (Figure 1B). When used solely in the activation stage, EC144 decreased the percentage of speck formation to 50%, compared with a 74% reduction when added together with LPS (Figure 1B). By comparison, the well‐reported NLRP3 inhibitor MCC950 completely blocked speck formation when added before or after LPS (with similar IC_50_ ~90 nM) consistent with its effect as a direct inhibitor of NLRP3 ^27^ (Figure 1C). These data suggest that while EC144 partially inhibits NLRP3 activation, some of its activity in the above and subsequent experiments must be attributed to a significant effect on the LPS priming response. We subsequently studied the effects of EC144 on caspase‐1 activation, IL‐1β secretion and pyroptotic cell death (LDH release) when dosed at the same time as LPS. Under these conditions, EC144 inhibited LPS and nigericin induced caspase‐1 activation with an IC_50_ of 150 nM (Figure 1D). EC144 was then used at a single dose (10 µM) in the same experimental paradigm to test its effects on IL‐1β secretion and cell death. Under these conditions, EC144 inhibited both IL‐1β release (Figure 1E) and cell death (Figure 1F).

The effects of EC144 (1 μM) on LPS‐induced priming were then assessed in primary mouse BMDMs. Primary BMDMs were incubated with EC144 for 15 min prior to a 4‐h incubation with either 100 ng ml^−1^ or 1 μg ml^−1^ LPS, after which lysates were harvested and analysed for expression of NLRP3, pro‐IL‐1β or HSP70, by Western blot (Figure 1G). Supernatants from this experiment were harvested and analysed for IL‐6 by ELISA (Figure 1H). Here, EC144 inhibited priming induced by either dose of LPS as expressions of NLRP3 and pro‐IL‐1β were inhibited (Figure 1G) as was production of IL‐6 (Figure 1H). Previously, anti‐inflammatory effects of HSP90 inhibition have been attributed to an induction of HSP70.^28^ However, here the effects of EC144 on the inflammasome cannot be attributed to increased levels of HSP70, as its levels did not change with EC144 treatment (Figure 1G).

We next assessed the potential for EC144 to inhibit NLRP3 inflammasome responses *in vivo*. To do this, we assessed its effects in an *in vivo* model of peritonitis as described previously.^17^, ^29^ Briefly, mice were orally gavaged with vehicle, or 10, 20 or 30 mg kg^−1^ EC144, or 20 mg kg^−1^ of the NLRP3 inhibitor MCC950 and then dosed with LPS (1 µg) i.p. Two hours later, animals were anaesthetized and injected i.p. with ATP (100 mM, 500 µl in PBS) for 15 min before peritoneal lavage, and bloods and brains were taken for pharmacokinetic (PK) analysis. Lavage fluid was analysed for IL‐1β and another pro‐inflammatory cytokine IL‐6. EC144 at all doses completely inhibited inflammasome‐dependent IL‐1β release, comparable to MCC950 (Figure 2A). IL‐6 levels were also reduced by EC144, whereas MCC950 had no effect on IL‐6 (Figure 2B). PK analysis of the bloods and the brain showed significant plasma and brain levels of EC144 after oral gavage (Figure 2C). Given that all doses of EC144 had worked equally well, we repeated the experiment with 0·1, 1 or 10 mg kg^−1^ and again measured IL‐1β and IL‐6 levels. When dosed at 0·1 or 1 mg kg^−1^, EC144 was ineffective in this model, suggesting 10 mg kg^−1^ is the minimal effective inflammasome‐inhibiting dose *in vivo* (Figure 2D,E).

There is great interest in the development of inhibitors for the NLRP3 inflammasome. We report here the potential for targeting HSP90, which is known to regulate NLRP3 activation and IL‐1β secretion.^14^, ^15^, ^16^ Clearly targeting HSP90, a highly abundant protein with many interactions that include innate immune signalling pathways,^13^ will not provide high specificity for NLRP3. Indeed, EC144 is known to inhibit systemic TNF‐α production in LPS‐injected mice, and is protective in a mouse collagen‐induced arthritis model.^30^ Our data (Figure 2) also show that in addition to inhibiting NLRP3‐dependent IL‐1β production, EC144 also significantly attenuated production of IL‐6 *in vitro* and *in vivo*. HSP90 regulates the activity of a number of proteins involved in the proliferation of tumour cells and for this reason is considered a therapeutic target in cancer. The HSP90 inhibitor BIIB021 has completed both phase I^21^ and phase II^31^ clinical trials as a potential cancer therapy and so its PK and safety profile in humans are well established. It is possible that HSP90 inhibitors would make effective therapeutics for inflammatory disease, especially acute disease. For example, severely ill patients with COVID‐19 are characterized by a hyper‐inflammatory state, with high levels of IL‐6.^32^ Given there are currently clinical trials underway with drugs to target IL‐6 and IL‐1 in COVID‐19, in addition to other immunomodulatory agents, it is possible that an orally active and rapidly acting anti‐inflammatory drug will be of value to conditions characterized by hyper‐inflammation.

## AUTHOR CONTRIBUTIONS

John B. Davis, Elena Di Daniel and David Brough conceptualized the study. Sohaib Nizami, Kanisa Arunasalam, Jack Green, James Cook, Catherine B. Lawrence and Tryfon Zarganes‐Tzitzikas contributed to methodology. Sohaib Nizami, Kanisa Arunasalam, Jack Green, James Cook, Catherine B. Lawrence and Tryfon Zarganes‐Tzitzikas underwent investigation. John B. Davis and David Brough collected resources. John B. Davis, Elena Di Daniel, and David Brough underwent supervision. John B. Davis, Elena Di Daniel, and David Brough contributed to funding acquisition. All authors contributed to writing the original draft, and writing, reviewing and editing of the manuscript. We would like to acknowledge Pharmidex for performing the PK analysis. We thank Val Millar and Daniel Ebner from the Target Discovery Institute for providing image analysis expertise and access to high content imaging instrumentation.

## FUNDING INFORMATION

The work carried out in the Oxford Drug Discovery Institute was supported by ARUK grant (ARUK‐2015DDI‐OX). This work was also supported by MRC grant MR/T016515/1 to DB and by a Presidential Fellowship (University of Manchester) to JG.

## CONFLICT OF INTEREST

The authors declare no conflict of interest.

## ETHICAL APPROVAL

All procedures were performed with appropriate personal and project licences in place, in accordance with the Home Office (Animals) Scientific Procedures Act (1986), and approved by the Home Office and the local Animal Ethical Review Group.

## Data Availability

The data that support the findings of this study are available from the corresponding author upon reasonable request.
